# Collective Chasing Behavior between Cooperators and Defectors in the Spatial Prisoner’s Dilemma

**DOI:** 10.1371/journal.pone.0067702

**Published:** 2013-07-05

**Authors:** Genki Ichinose, Masaya Saito, Shinsuke Suzuki

**Affiliations:** 1 Systems and Control Engineering, Anan National College of Technology, Anan, Tokushima, Japan; 2 JSPS fellow, Graduate School of Letters, Hokkaido University, Sapporo, Hokkaido, Japan; 3 Laboratory for Integrated Theoretical Neuroscience, Riken Brain Science Institute, Wako, Saitama, Japan; 4 Division of Humanities and Social Sciences, California Institute of Technology, Pasadena, California, United States of America; Umeå University, Sweden

## Abstract

Cooperation is one of the essential factors for all biological organisms in major evolutionary transitions. Recent studies have investigated the effect of migration for the evolution of cooperation. However, little is known about whether and how an individuals’ cooperativeness coevolves with mobility. One possibility is that mobility enhances cooperation by enabling cooperators to escape from defectors and form clusters; the other possibility is that mobility inhibits cooperation by helping the defectors to catch and exploit the groups of cooperators. In this study we investigate the coevolutionary dynamics by using the prisoner’s dilemma game model on a lattice structure. The computer simulations demonstrate that natural selection maintains cooperation in the form of evolutionary chasing between the cooperators and defectors. First, cooperative groups grow and collectively move in the same direction. Then, mutant defectors emerge and invade the cooperative groups, after which the defectors exploit the cooperators. Then other cooperative groups emerge due to mutation and the cycle is repeated. Here, it is worth noting that, as a result of natural selection, the mobility evolves towards directional migration, but not to random or completely fixed migration. Furthermore, with directional migration, the rate of global population extinction is lower when compared with other cases without the evolution of mobility (i.e., when mobility is preset to random or fixed). These findings illustrate the coevolutionary dynamics of cooperation and mobility through the directional chasing between cooperators and defectors.

## Introduction

Cooperation is one of the essential factors for all biological organisms in major evolutionary transitions. Cooperation to help others incurs some cost to the actor, and natural selection therefore favors a more selfish behavior unless a specific mechanism is introduced. Several mechanisms have been proposed for the evolution of cooperation [1]: kin selection [2], direct reciprocity [3], indirect reciprocity [4], network reciprocity [5], and multilevel (group) selection [6]. For each mechanism, the inherent principle is different; however, one very common feature is that cooperators tend to interact more with themselves than with defectors. This significantly contributes to the evolution of cooperation.

Given that biological interactions in the real world are often local rather than global, researchers have investigated the evolution of cooperation on networks such as square lattices [7], [8], small-world [9], [10] or scale-free networks [5], [11], [12]: individuals in a population occupy the nodes of the network, and the links define who interacts with whom. Since the seminal work by Nowak and May [7], numerous studies have demonstrated that the network structures facilitate the evolution of cooperation by allowing cooperators to form clusters with each other; this is termed as network reciprocity [1].

For example, Santos and Pacheco [5] have shown that a scale-free network structure, in which the number of links for each individual is highly heterogeneous, can facilitate network reciprocity (but see Masuda [13]). Moreover, Wang et al. [14], [15] have investigated the effect of network density by employing a square lattice with empty sites. They have found that there is an optimal density for the evolution of network reciprocity in the sparse environments. Furthermore, recent studies have specified various factors such the heterogeneity in the way of adopting the fittest strategy [16], [17], age structure [18], teaching activity [19], inferring reputation of individuals [20], and the diversity in the mapping of game payoffs to individual fitness [21] that promote cooperation on a network. Taken together, it has been shown that local interactions of individuals make it possible for cooperators to form clusters with each other and then enable the evolution of cooperation, which is facilitated by various types of heterogeneity. However, it is unknown how cooperators form clusters with other distant cooperators. In other words, we desire to know if they are able to escape from defectors while maintaining the cluster.

The migration of individuals is one of the effective mechanisms for cooperators to generate such assortative interactions with themselves rather than with defectors. Interest in the effect of migration has gradually increased, as shown in recent studies [22]–[43]. Some studies have assumed that migration occurs randomly, which is known as “non-contingent” migration [22], [24], [28]–[31], [36], [42]. The effect of such random migration on cooperation has been intensively explored by Vainstein et al. [31]. They have assumed that each individual moves to a randomly chosen site in a random timing within its four nearest neighbors in a square lattice environment. It has been found that cooperation is promoted at the intermediate mobilities and densities compared with the no migration case. This model has also been extended to various games (such as snowdrift and stag hunt) on a spatial structure [36].

Animals often migrate to other places conditionally, and not randomly. For instance, Jiang et al. [41] have introduced adaptive migration in spatial games. In their model, an individual moves to an empty site with a 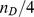 probability, where 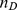 is the number of defectors in the four adjacent neighbors. They have shown that this type of migration promotes cooperation, especially in the intermediate density. In the model by Yang et al. [40], aspiration-induced migration has been assumed, in which an individual moves to a randomly chosen empty site within its four neighbors if the payoff for the individual falls below his aspiration level. The optimal level of cooperation has been found when both the aspiration level and the density are in the medium range. Moreover, Helbing et al. [35], [37] have shown that in addition to the timing of migration, cooperation is facilitated if individuals can move to their preferred destination. Despite the significant effect of contingent movement on the evolution of cooperation, one caveat to these studies is that a heightened awareness of the environment is often required for this type of movement. For more basic biological organisms such as cells, it is hard to detect whether their current location is good or bad. Furthermore, they cannot know for sure if the destination to which they are moving is good or bad. For such organisms, a more primitive rule for the movement is plausible.

Here, it is worth noting that most of these studies on the effect of migration have treated migration as an exogenous non-evolvable trait (but see [27], [28], [42]). However, in the real world, the rate and directionality of the migration must evolve under natural selection. In other words, the following issues cannot be addressed in the previous studies. Imagine that in an ecosystem, some individuals migrate to the right very quickly, others migrate slowly to the left, and the remainder never migrate. Which types spread over the population as a result of natural selection? How does the evolution of migration interact with the evolution of cooperation?

In this study, we investigate the coevolution of migration and cooperation. To address this issue, we have constructed a spatial prisoner’s dilemma (PD) game model in which each individual’s cooperativeness, and the migration rate, and direction can be either enhanced or suppressed by natural selection. Notably, our model differs from those incorporating contingent migration in that the individuals’ high intelligence is not postulated. For example, the timing of migration depends solely on the inherent rate, but not on the environmental situations (e.g., the neighbors’ cooperativeness, *etc.*) and each individual does not necessarily know beforehand whether or not the destination of the migration is favorable. In this sense, we believe that the present model is applicable to the coevolution of cooperation and mobility in a wide range of organisms in the real world.

### Spatial PD Model with Directional Evolution

**Figure 1:**
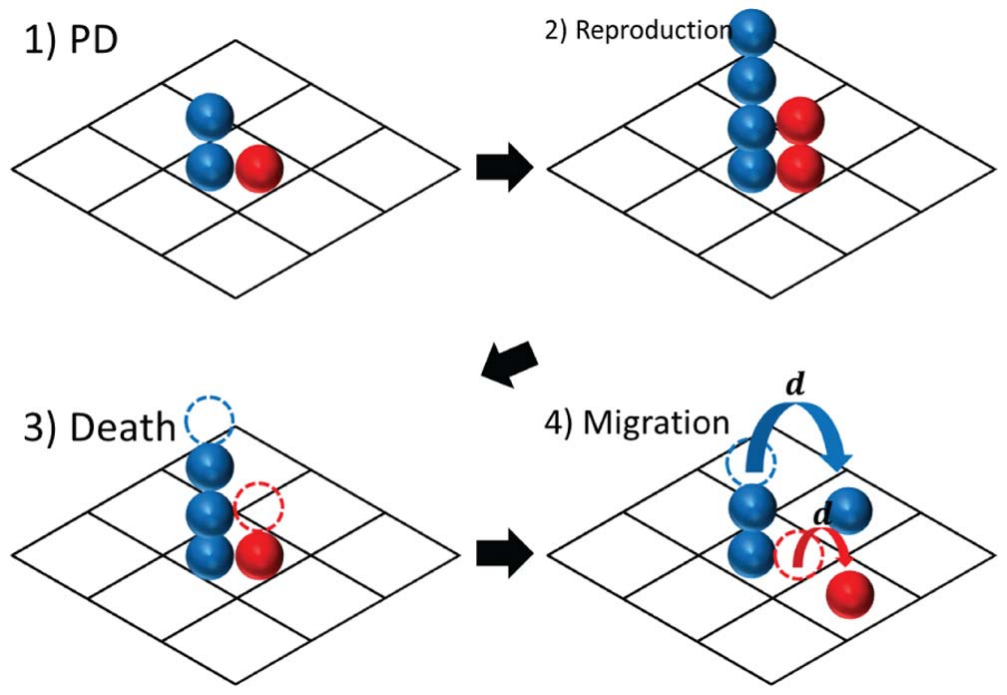
Spatial PD model in a two-dimensional square lattice with directional evolution. Blue (Red) represents a cooperator (defector). The simulation flow is as follows: 1) Each individual plays PD with others within the same site. 2) Each individual reproduces one offspring with a certain probability (see **Models**). 3) Each individual dies with a probability 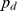. 4) Each individual migrates to one of four neighbors with a probability depending on 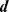. These four steps are then repeated.

We consider a model in which 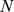 initial individuals play PD, reproduce, die, and migrate, all in a two-dimensional square lattice. The direction of migration is determined in accordance with a probability vector 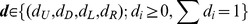 in which each element indicates the weight of the movement to an upper space (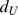), a down space (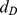), a left space (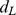), and a right space (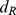). The PD strategy, migration rate, and this probability vector evolve under natural selection (see details in Table 1). The flow of the simulation is described in Fig. 1, and **Models** presents a detailed setting. The evolutionary simulations identify interesting phenomena: cooperators collectively move in a particular direction; next, defectors chase the cooperators, and after which the defectors catch up with the cooperators, and the local population almost becomes extinct; other cooperative clusters emerge from defectors due to mutation. After returning to the initial state, similar cycles are then repeated. We find that the oscillatory cooperation and defection dynamics are balanced as a result of this chasing behavior, which results in population stability.

**Table 1:** Parameters in basic settings.

Each individual’s strategy	
(evolved by natural selection)	
Cooperativeness	*C* or *D*
Migration rate	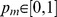
Direction of the migration	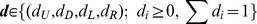
**Environment**	
Size of the lattice	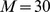
Initial population size	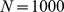
Benefit-to-cost ratio of cooperation	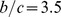
Probability of death	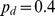
Mutation rate of strategy	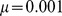
Variance of the mutation in 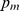 and ***d***	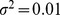

## Results

**Figure 2:**
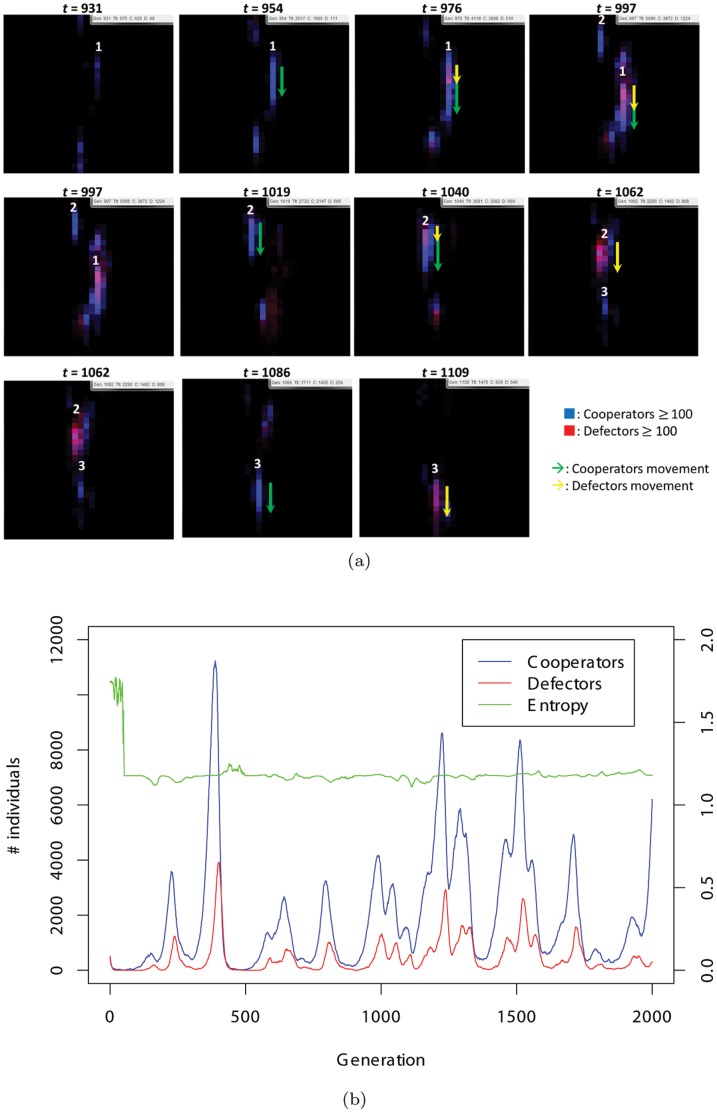
(a) Typical snapshots of the downward evolution in Fig. **2(b).** Blue represents cooperators and red represents defectors. If there are less than 100 of each type, each color (blue or red) is reduced gradually. If there are two types of individuals in the same site, the colors are mixed. The green lines indicate the movement of cooperators, and yellow lines indicate the movement of defectors. The numbers next to each group are labels. The box located at the top-right space in each square indicates the generation, number of cooperators, and number of defectors. After cooperative clusters emerge and move downward, they are chased and exploited by defectors. However, other cooperative clusters emerge, and then these cycles are repeated. Video S1 shows a series of this evolution. (b) Dynamics of the number of individuals with entropy (explained later). Blue signifies cooperators and red signifies defectors.

Evolutionary simulations of the model show the coevolutionary dynamics of “cooperativeness” and “mobility toward a particular direction (directional migration),” which we call evolutionary chasing between cooperators and defectors. A typical dynamics is as follows (see Fig. 2(a)). At the initial generation, clusters of cooperators emerge in several spaces by chance. Meanwhile, some of the cooperative clusters remaining in the same locations are exploited by defectors. In contrast, the other clusters can flourish by moving in a specific direction. However, the success of cooperators does not last long because mutated defectors move in the same direction with cooperators and invade them. The local populations are therefore almost extinct, but new cooperators suddenly emerge again possibly due to mutation (examined later). These cycles spontaneously arise in various times and spaces.

Figure 2(a) shows snapshots of a typical evolutionary dynamics (see also Video S1 for the first supporting information video). In this example run, the evolution of migration is almost vertical. In the 931st generation, a cooperative cluster is generated (labeled “1”), which then spreads to a lower space because they are mutually beneficial to each other (954th). The cooperators continue to move in the same direction, while some defectors emerge in the center of the cluster (976th). In the 997th generation, the defectors move in the same direction as the cooperators and invade them, as a result of which the cooperators are almost all exploited (997th). In the same generation, another cooperative cluster is generated at the top space (labeled “2”). They move in a downward direction (1019th and 1040th) but are exploited by defectors in the 1062nd generation. As a result, the #2 population collapses (1086th). The #3 population then repeats the same cycle. In this run, such vertical movements of the individuals are frequently observed (see Fig. S1 in File S1).

**Figure 3:**
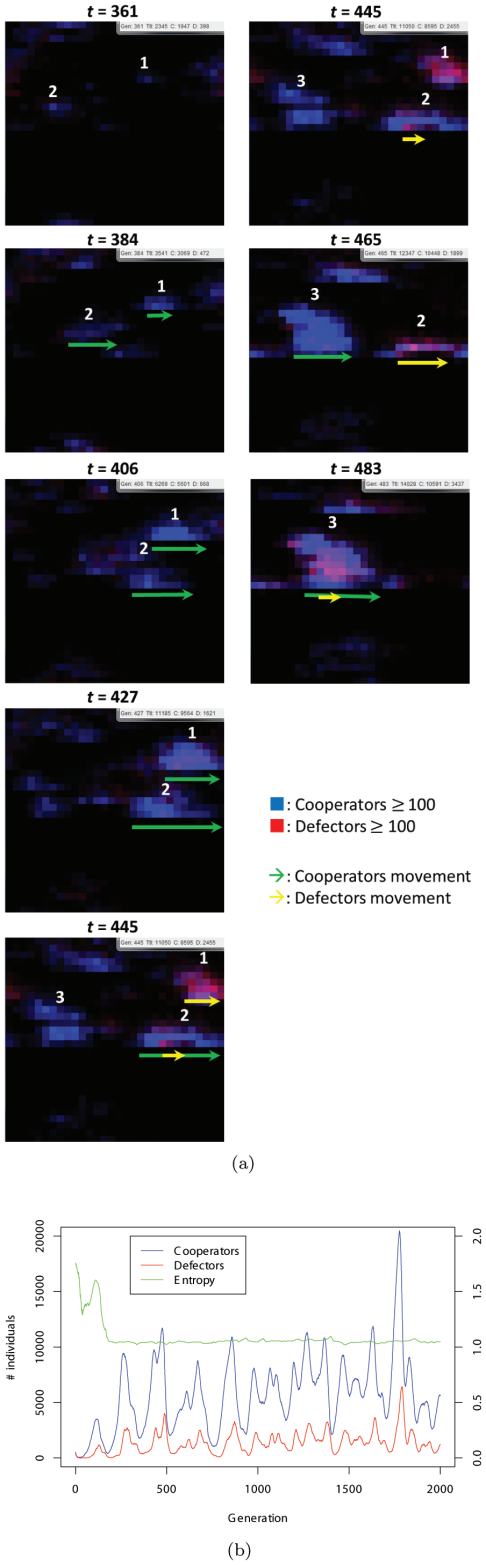
(a) Typical snapshots showing rightward evolution. Note that in this case, the time axis is vertical. Also see Video S2. (b) Dynamics of the number of individuals with entropy (explained later) in the evolution to the right.

In another simulation run, we find the evolution of a different direction in which the horizontal migrations of the individuals are observed (Fig. 3(a)). Video S2 shows a series of this evolution. Oscillatory dynamics are also observed during this cycle; however, in this case, evolution results in a directional migration to the right. In the 361st generation, two different cooperative groups (labeled “1” and “2”) emerge and move to the right while increasing their peers (384th, 406th, and 427th). In the 445th generation, the #1 cooperative group is invaded by defectors, while a new group emerge (labeled “3”). Eventually, the #2 and #3 groups are exploited by the defectors (465th and 483rd, respectively). We observe chasing behaviors common throughout the simulation runs, although the direction is different for each evolutionary dynamics.

### Comparison with Two Extreme Cases

**Figure 4:**
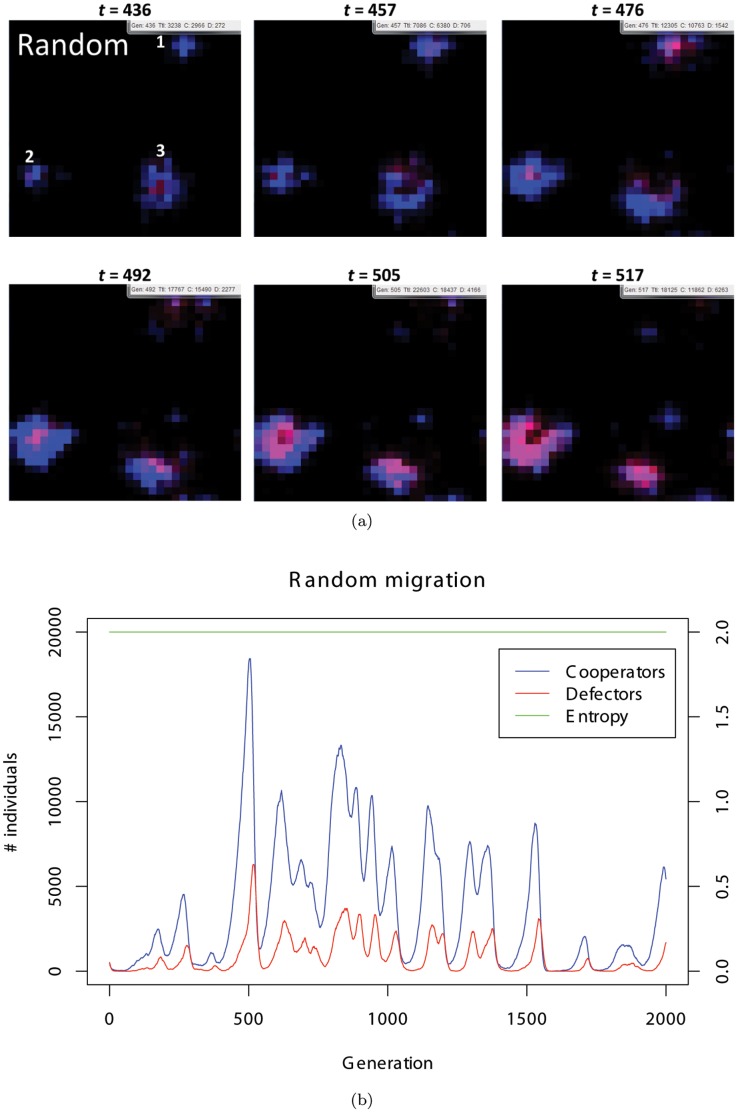
(a) Typical snapshots of the random migration. (b) The population dynamics of random migration shown in (a). The entropy is 2.0 according to the definition.

**Figure 5:**
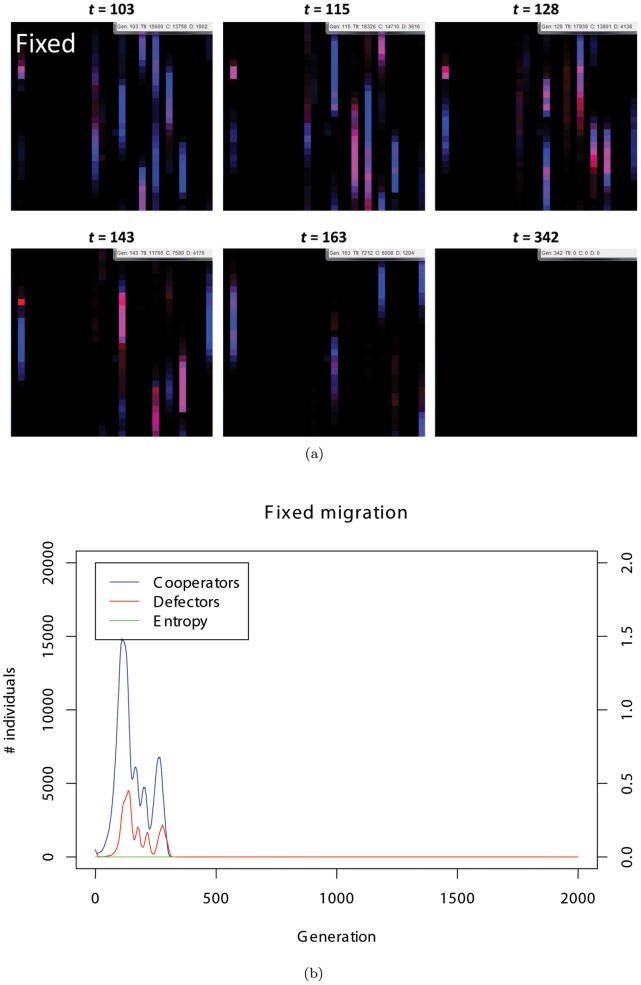
(a) Typical snapshots of the single fixed migration. The individuals’ movements are limited to the upward direction. (b) The population dynamics of fixed migration shown in (*a*). The entropy is 0.0 according to the definition.

We next try to understand the nature of the chasing behavior by comparing our results with the two extreme cases: random and fixed migration. In the random migration model, all the individuals move in a random direction, that is, their probability vectors are fixed at 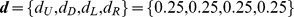 for all individuals through a simulation (Fig. 4(a)). On the other hand, in the fixed migration model, all the individuals move in the same direction (an upward direction), i.e., the probability vector is fixed at 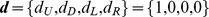 for all individuals throughout the simulation (Fig. 5(a)).

**Figure 6:**
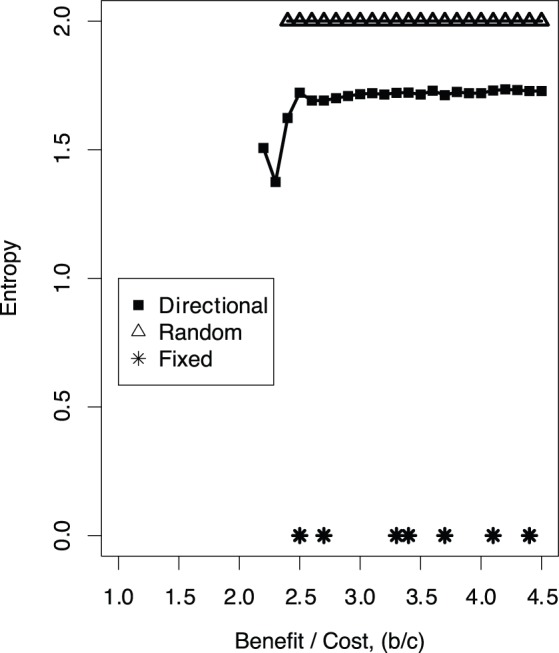
Entropy from the 1000th to 2000th generations (averaged over 1000 simulation runs, excluding the runs resulting in extinction) as a function of the benefit-to-cost ratio of cooperation in the three models.

First, we quantitatively show that in our model, natural selection favors directional migration, and not random or fixed migration. The directionality of the migration is represented by the degree to which elements in the probability vector, ***d***, are biased. This can be measured by entropy (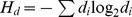). In our main model, by natural selection, the average value of the entropy over individuals results in between *zero* and *two* regardless of the benefit to cost ratio of cooperation (see green lines in Fig. 2(b) and 3(b) for the typical dynamics shown in the previous section; Fig. 6 shows the averaged values over 1000 runs of the simulation as a function of the benefit to cost ratio of cooperation). Given that the value of entropy is by definition *two* in random migration and *zero* in fixed migration, these results indicate that natural selection leads individuals to move in a particular, but not completely the same, direction accompanied by the chasing between cooperators and defectors.

**Figure 7:**
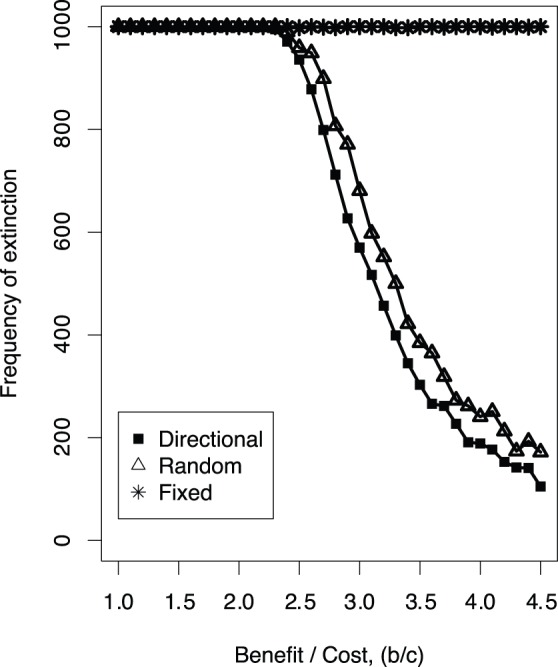
Frequency of extinction (over 1000 simulation runs) as a function of the benefit-to-cost ratio of cooperation.

**Figure 8:**
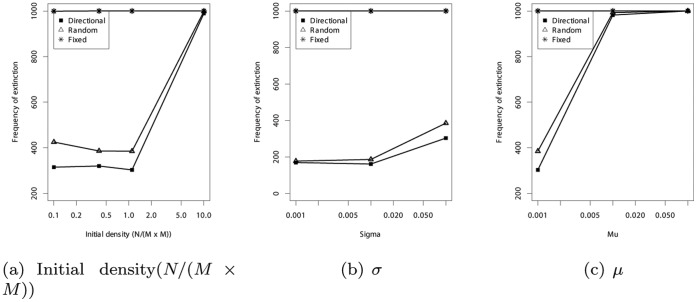
Frequency of extinction (over 1000 simulation runs) as a function of the (a) initial density (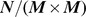), (b) 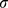, and (c) 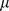. (a) 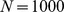 is the initial number of individuals. The square lattice is composed of 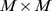, and only 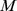 takes values of 10, 30, 50, and 100. Thus, 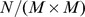 means the initial density. (b) 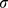 is the SD of the normal distribution in the mutation of 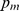 and ***d***. (c) 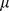 is the mutation rate. In all cases, the directional migration is the lowest extinction rates. Other results as functions of these parameters have been described in Fig. S2, S3, and S4 in File S1.

Second, we demonstrate the difference in the frequency of extinction between our original model and random/fixed migration models. In the fixed migration model, evolutionary dynamics results in the rapid extinction (see Fig. 5(b) for a typical example). Sensitivity analyses based on the average extinction rate of over 1000 simulation runs show that this statement holds true irrespective of the benefit to cost ratio of cooperation (see Fig. 7), initial density of the individuals (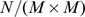; see Fig. 8(a)), variance of the mutation (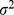; see Fig. 8(b)), or rate of the mutation (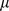; see Fig. 8(c)). This indicates that flexibility in the direction of migration is essential to sustain cooperation.

In the random migration model, evolutionary dynamics initially appears to be similar to that in the original model, that is, the oscillation of the frequency of cooperators and defectors (see Fig. 4(b) and Fig. 2(b)). However, an examination of the average extinction rate over 1000 simulation runs reveals that the rate is greater in the random migration model compared with that in our original model. We further confirm that this difference is observed in a wide range of parameter settings such as the benefit to cost ratio of cooperation, initial density of the individuals, rate of the mutation or variance of the mutation (see Figs. 7 and 8). Taken together, these extinction rate results suggest that a moderate level of flexibility in the direction of migration (not fixed or complete random migration), facilitates the maintenance of cooperation as a form of evolutionary chasing between cooperators and defectors.

### Role of Mutation in the Chasing Behaviors

We hypothesize that mutation plays a key role in the chasing behaviors between cooperators and defectors. More specifically, mutation would be critical for the spontaneous emergences of new cooperative clusters after the existing cooperative clusters are almost exploited by defectors.

To test the hypothesis, we investigate the evolutionary dynamics in the absence of the strategy mutation (i.e., 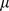 = 0; the other settings are the same as the original model). Simulation results show that evolutionary chasing behavior is never observed in the absence of mutation. Cooperators diverge to infinity (If the global population reaches 100,000, it is defined as divergence) (Video S3) or the global population goes extinct. From 100 independent simulation runs, we observe 89 instances of the former case and 11 instances of the latter. This result shows the critical role of mutation in the maintenance of the chasing behavior.

Moreover, we conduct another control experiment in the absence of mutation. In this model, all of the 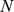 individuals are initially set up at the same site (Fig. S6, S7 in File S1, Video S4, S5, and S6), and the population consists of only one defector and the other 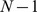 cooperators. As well as the previous control model, the chasing behavior is never observed, and the simulation displays two patterns of the evolutionary dynamics, the cooperator divergence (Fig. S6, Video S4), and the defection invasion (Fig. S7 in File S1, Video S5 and S6). In the case of invasion, a cooperative cluster grows up at the center in early generations; next, the cluster is gradually invaded by defectors from the inner region. Interestingly in this case, unlike the original case (see Fig. 2(a)), new clusters of cooperators never appear after the first rise of cooperators. This finding further supports our hypothesis that mutation plays a key role in the chasing behaviors between cooperators and defectors, especially in the spontaneous emergences of new cooperative clusters.

## Discussion

Many organisms are mobile. They move around to find food or to escape from harsh environments [44]. Moving around is also important for collectively interacting with peers and/or for forming a cooperative relationship with new individuals. In the present study, we have investigated the coevolutionary dynamics of cooperativeness and mobility (rate and direction of migration). Computer simulations have demonstrated that natural selection maintains cooperation as a form of oscillatory chasing between cooperators and defectors and that mobility evolves to the directional migration. Furthermore, we have found that the population extinction rate in the coevolutionary dynamics is lower when compared to the case without the evolution of mobility (directionality of migration is preset to random or fixed). These findings exhibit the coevolutionary dynamics of cooperation and mobility through the oscillatory chasing between cooperators and defectors with directionality.

Recently Suzuki and Kimura [42] have shown that the number of cooperators and defectors is indicative of the oscillatory dynamics in the coevolution of cooperation and mobility. However, the underlying mechanism remains elusive. In this study, we have demonstrated that chasing between cooperators and defectors with directionality results in the oscillatory dynamics and the coevolution of cooperation and mobility. In the oscillatory dynamics, cooperative groups grow in certain local spaces and collectively move in the same direction (i.e., directional migration). Next, mutant defectors arise by chance in the groups, and then exploit the cooperators. The cooperative groups therefore collapse, but other cooperators emerge due to mutation in different spaces. The cycle is then repeated. To our knowledge, this is the first study to reveal the mechanism underlying the coevolution of cooperation and mobility.

Although previous studies have focused mainly on the mechanism by which cooperation is evolutionarily stable, there has been concern expressed recently that cooperation can be maintained as a dynamical attractor, such as the oscillation or chaos of the evolutionary system [45]–[47]. Here, we have shown such a form of the evolution of cooperation in which cooperation is maintained by escaping from the cashing of defectors in various times and spaces.

The importance of directional migration is highlighted by comparing the two extreme cases: fixed and random migration. First, we have quantitatively shown that natural selection leads to directional migration, and not fixed or random migration. Second, under the directional migration favored by natural selection, the population extinction rate is lower than that in the random and fixed migration case. These results can be interpreted as follows. In the case of fixed migration, it is impossible for cooperators to flexibly move around and escape from defectors. On the other hand, in the random migration case, cooperators cannot collectively move in the same direction to form clusters. Consequently, the evolution of cooperation is more likely to be accomplished under directional migration. In summary, we have discovered that mobility evolves to the directional migration with the lowest rate of extinction, suggesting the importance of appropriate flexibility in the direction of migration for the evolution of cooperation.

We can consider other types of the interaction networks such as triangle lattice, honeycomb, kagome, scale-free network, and small-world network. It has been known in addition to the number of neighbors, various aspects of the network structure (e.g., clustering coefficient) affect the evolution of cooperation. For example, Perc et al. [48] have shown that on the square and honeycomb lattices (clustering coefficient = 0), an intermediate level of noise in the strategy adoption is optimal for the evolution of cooperation, while the strategy adoption without noise is optimal on kagome and triangle lattice (clustering coefficient>0). One possible extension of this study is to examine the existence of chasing behaviors, even in other interaction structures.

In the present study, we have not assumed individuals’ high cognitive abilities, in contrast to the previous studies regarding the effect of migration on the evolution of cooperation [23], [25], [32]–[35], [37], [43]. For example, the timing of migration depends solely on the inherent rate, but not on the environmental situations (e.g., the neighbors’ cooperativeness, *etc.*) and each individual does not necessarily know beforehand whether or not the destination of the migration is favorable. In this sense, we believe that our model can be applied to the emergence of cooperation in a wide range of scales, including molecules, cells, organisms, ecosystems, and human societies.

In this study we have shown the coevolution of cooperation and directional migration, which reminds us of the historical story of the humans who migrated out of Africa. It has been believed that humans migrated from the African continent to the European and Asian continents about 45–60 thousand years ago [49]. They collectively moved and helped each other along the way. Our study would provide insights into the evolutionary basis of the great human migration. That is, natural selection encouraged them to move correctively and cooperate with each other.

### Models

Here, we describe the details of the model used, which is extended from that used by Suzuki and Kimura [42]. The environment is a two-dimensional 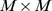 square lattice (each of the four edges is connected to the opposite one). Each lattice-site can be occupied by one or more individuals and can be vacant. At the beginning of each generation, each individual plays an 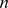-person prisoner’s dilemma game [50]–[52] in each site. In this game, the cost and benefit of cooperation are denoted as 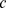 and 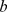, respectively, where 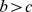, and the benefit is shared equally among the 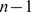 other individuals at the site. Thus, the payoffs for a cooperator, 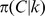, and that for a defector, 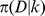, where 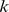 is the number of others cooperating in the site (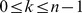), are given as
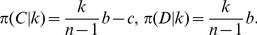
(1)


If there is only one individual in a site, the payoff is presumed to be 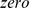, following which each individual leaves an offspring at the same site depending on the payoff received in that particular generation. With this process, a higher payoff implies that there is a higher probability that the individual will leave an offspring. More specifically, let 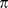 be the payoff of an individual with a probability of 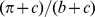 that one offspring will be left. This indicates that the probability linearly increases as the payoff increases (note that in theory, 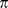 varies from 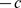 to 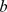 in the prisoner’s dilemma game). Moreover, the probability that each individual dies is 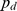, irrespective of the payoff received. The evolutionary process is controlled by the balance between the probability of the reproduction and that of the death. In our model, we assume that each individual’s reproduction depends on his/her fitness (payoff of the game) while the death probability, 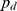, is constant. Note that each individual’s “total” reproduction performance depends on the fitness, and is hence consistent with the concept of natural selection. An extremely large death probability can lead to population extinction. Conversely, if the probability is extremely small, the population size diverges to infinity. Both are unrealistic situations in the real world. We therefore set the death probability at an intermediate value, i.e., 0.4 (Table 1). See also the sensitivity analysis of the death probability (Fig. S4 in File S1). In essence, each offspring inherits the parent’s strategy, that is, the degree of cooperation (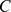 or 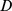), migration rate, 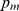, and direction weights, 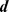. Thus, these three parameters are our main focal variables, which evolve under natural selection (Table 1). In addition, mutation is introduced: with a small probability 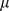, a cooperator becomes a defector, and vice versa. The migration rate, 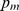, and the randomly selected 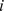
th element of the probability vector, 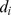, change to 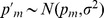, 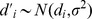. If 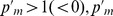 is again set to be 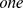 (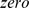), and 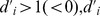 is again set to be 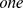 (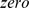). After that, 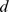 is again normalized to satisfy the property of the probability 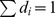. Finally, each individual moves to one of the four neighboring sites (i.e., von Neumann neighborhood) with weighted probability 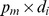 where 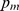 is the migration rate for each individual and 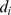 is the 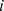
th element of the probability vector 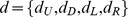, 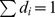. Each element of this vector indicates the weight of the movement to an upper space (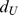), a down space (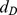), a left space (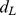), and a right space (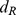).

In the first generation, 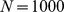 individuals are randomly distributed on the square lattice, and their strategies that indicate the degrees of cooperation, migration rate, and direction weights are randomly determined, while 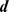 is normalized to satisfy 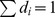. In this paper, we used the following parameter values as the basic settings unless mentioned otherwise: lattice size 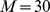, benefit-to-cost ratio of cooperation 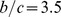, and mutation parameters 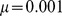 and 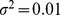. The parameter description is shown in Table 1.

## Supporting Information

File S1 **Supplementary materials.**
(PDF)

Video S1 (WMV)

Video S2 (WMV)

Video S3 (WMV)

Video S4 (WMV)

Video S5 (WMV)

Video S6 (WMV)
