# Supplementary material for: Collective Chasing Behavior between Cooperators and Defectors in the Spatial Prisoner’s Dilemma
Source: PLoS One. 2013 Jul 5;8(7):e67702. doi: 10.1371/journal.pone.0067702 (PMC3702560; doi:10.1371/journal.pone.0067702)
Supplement: File S1 — Supplementary materials. (PDF) [file pone.0067702.s001.pdf]

Supplementary materials:

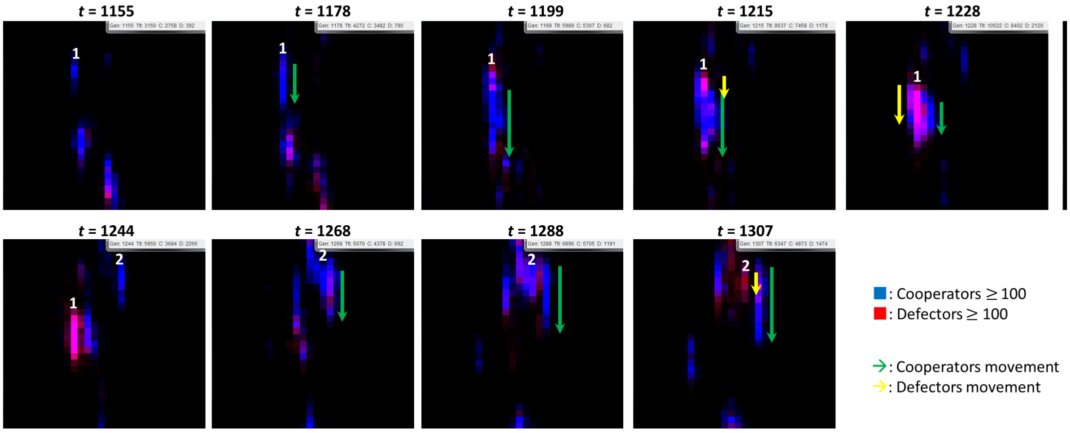

Figure S1 Other typical snapshots of the downward evolution in the same iteration as in Fig. 2. See also Video S1.

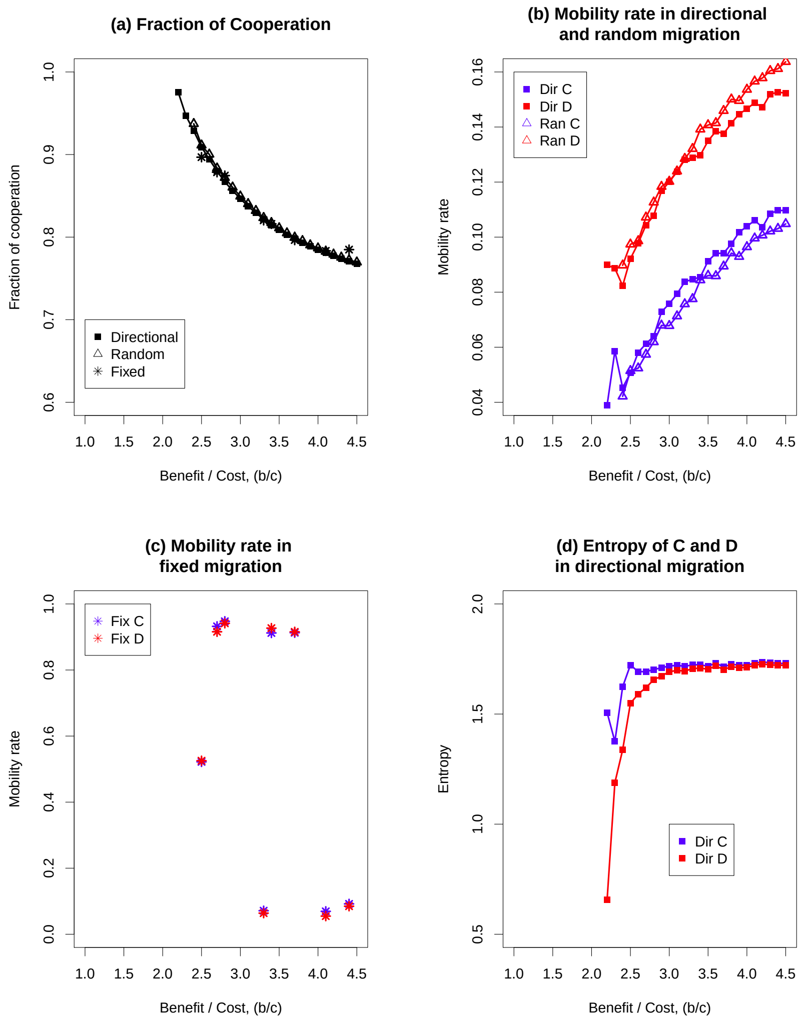

Figure S2 General analysis as a function of the benefit/cost ratio. (a) Fraction of cooperation from the 1000th to 2000th generation (averaged over 1000 simulation runs, excluding the runs resulting in extinction) as a function of the benefit-to-cost ratio of cooperation. (b) Mobility rates from the 1000th to 2000th generations (averaged over individuals and 1000 simulation runs, excluding the runs resulting in extinction) as a function of the benefit-to-cost ratio of cooperation in random and directional migration. (c) Mobility rate for fixed migration. (d) Average entropy of cooperators and defectors in directional migration.

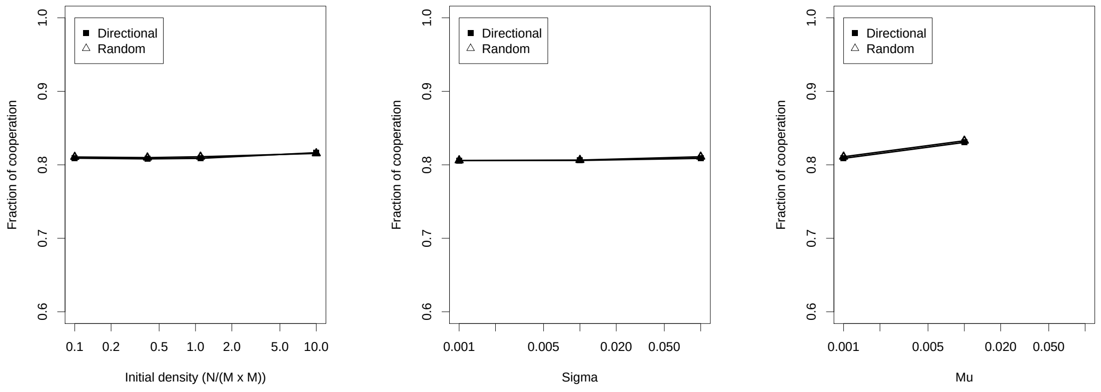

(a) Initial density$(N/(M \times M))$ (b) σ (c) μ

Figure S3 Sensitivity analysis. Fraction of cooperation (over 1000 simulation runs) as a function of the (a) initial density $(N/(M \times M))$, (b) σ, and (c) μ. The result of fixed migration is excluded because the extinction rate was almost 100% (See Fig. 8). (a) $N = 1000$ is the initial number of individuals. The square lattice is composed of $M \times M$, and only M takes values of 10, 30, 50, and 100. Thus, $N/(M \times M)$ denotes the initial density. (b) σ is the SD of the normal distribution in the mutation of p_m and $\boldsymbol{d}$. (c) μ is the mutation rate. At $\mu = 0.1$, all three models had died out (See Fig. 8).

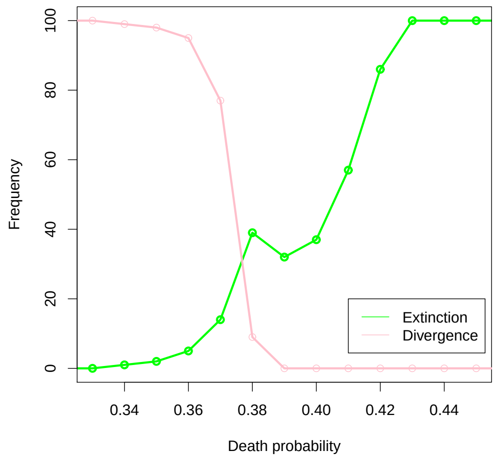

Figure S4 Sensitivity to death probability (p_d) in directional migration. We conduct 100 independent simulation runs. If the global population reaches 100,000, it is defined as divergence. At $p_d \leq 0.33 (\geq 0.43)$, all runs show divergence (extinction). Thus, $0.34 \leq p_d \leq 0.42$ is the realistic situation in which the finite global population is observed.

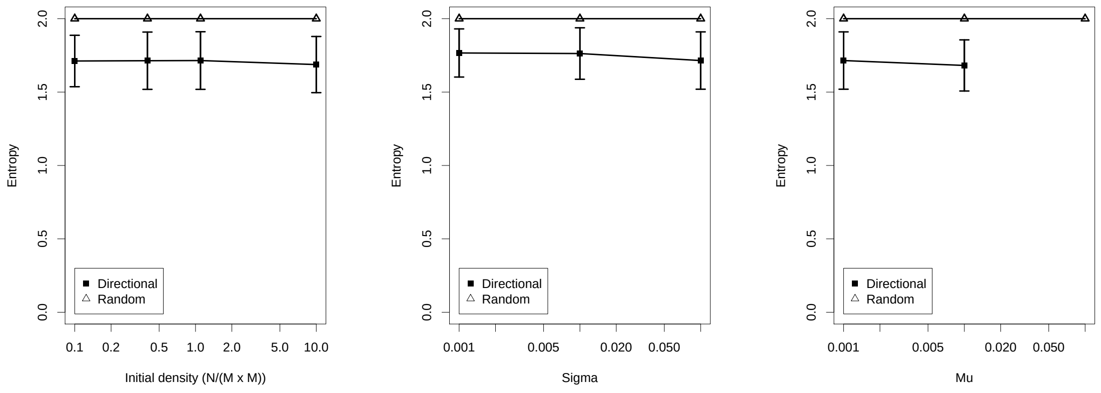

(a) Initial density$(N/(M \times M))$ (b) σ (c) μ

Figure S5 Entropy (over 1000 simulation runs) as a function of the (a) initial density $(N/(M \times M))$, (b) σ, and (c) μ with error bars (SD).

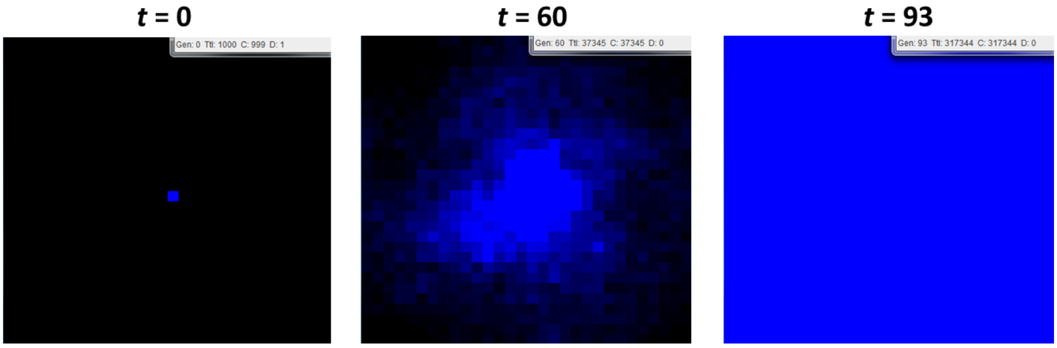

(a)

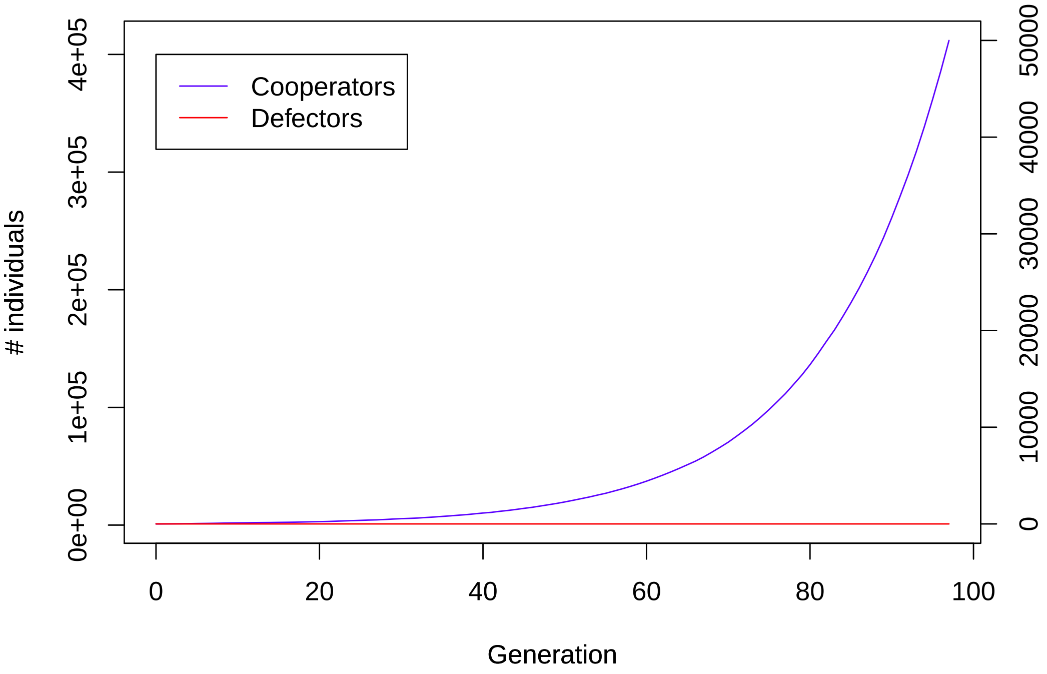

(b)

Figure S6 (a) Typical snapshots of cooperation divergence when individuals are allocated in the center with only one defector ($\mu = 0$). In this case, one defector does not expand and cooperators diverge quickly. After the 100th generation, the population is over 400,000. (b) The population dynamics of the cooperation divergence.

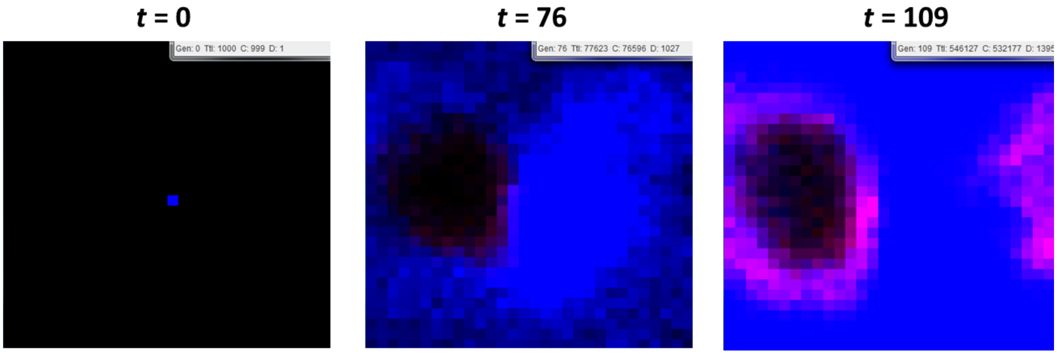

(a)

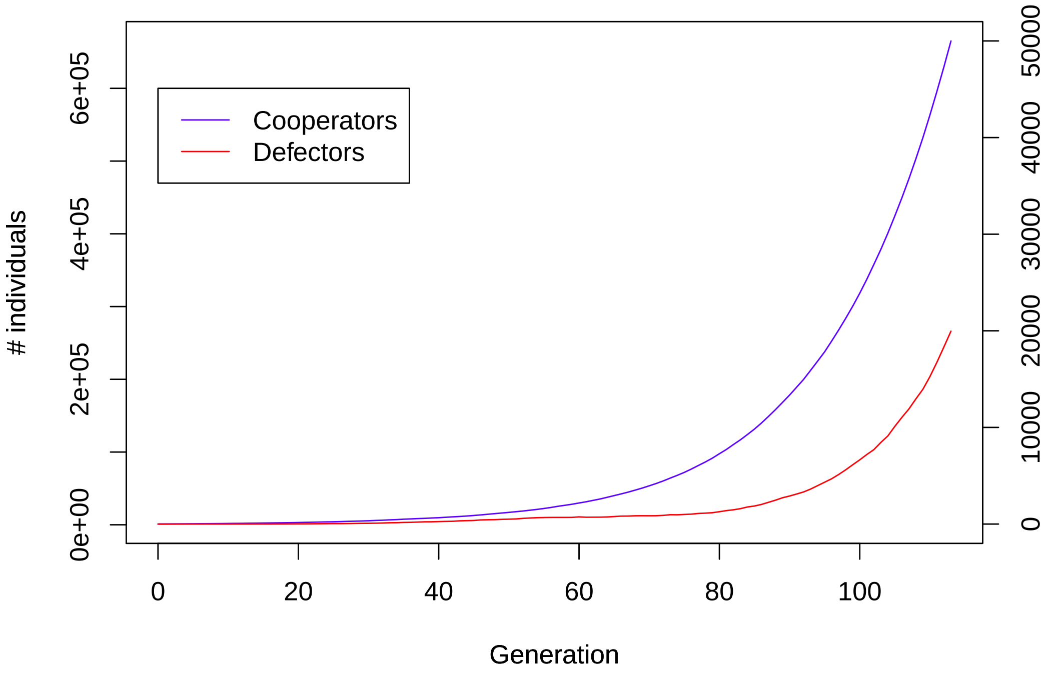

(b)

Figure S7 (a) Typical snapshots of defective invasion from the inner territory when individuals are allocated in the center with only one defector ($\mu = 0$). In this case, one defector invades cooperators, but they only survive at the border with cooperators. After the 100th generation, there are over 600,000 cooperators (left y-axis) and around 20,000 defectors (right y-axis). (b) The population dynamics of defective invasion.
